# Small Extracellular Vesicles and Metastasis—Blame the Messenger

**DOI:** 10.3390/cancers13174380

**Published:** 2021-08-30

**Authors:** Tanja Seibold, Mareike Waldenmaier, Thomas Seufferlein, Tim Eiseler

**Affiliations:** Department for Internal Medicine, University Clinic Ulm, 89081 Ulm, Germany; tanja.seibold@uniklinik-ulm.de (T.S.); mareike.waldenmaier@uniklinik-ulm.de (M.W.); thomas.seufferlein@uniklinik-ulm.de (T.S.)

**Keywords:** cancer, metastasis, small extracellular vesicles (sEVs), exosomes, immune evasion, organotropism, pre-metastatic niche

## Abstract

**Simple Summary:**

Due to their systemic nature, metastatic lesions are a major problem for curative cancer treatment. According to a common model for metastasis, tumor cells disseminate by local invasion, survival in the blood stream and extravasation into suitable tissue environments. At secondary sites, metastatic cells adapt, proliferate and foster vascularization to satisfy nutrient and oxygen demand. In recent years, tumors were shown to extensively communicate with cells in the local microenvironment and future metastatic sites by secreting small extracellular vesicles (sEVs, exosomes). sEVs deliver bioactive cargos, e.g., proteins, and in particular, several nucleic acid classes to reprogram target cells, which in turn facilitate tumor growth, cell motility, angiogenesis, immune evasion and establishment of pre-metastatic niches. sEV-cargos also act as biomarkers for diagnosis and prognosis. This review discusses how tumor cells utilize sEVs with nucleic acid cargos to progress through metastasis, and how sEVs may be employed for prognosis and treatment.

**Abstract:**

Cancer is a complex disease, driven by genetic defects and environmental cues. Systemic dissemination of cancer cells by metastasis is generally associated with poor prognosis and is responsible for more than 90% of cancer deaths. Metastasis is thought to follow a sequence of events, starting with loss of epithelial features, detachment of tumor cells, basement membrane breakdown, migration, intravasation and survival in the circulation. At suitable distant niches, tumor cells reattach, extravasate and establish themselves by proliferating and attracting vascularization to fuel metastatic growth. These processes are facilitated by extensive cross-communication of tumor cells with cells in the primary tumor microenvironment (TME) as well as at distant pre-metastatic niches. A vital part of this communication network are small extracellular vesicles (sEVs, exosomes) with a size of 30–150 nm. Tumor-derived sEVs educate recipient cells with bioactive cargos, such as proteins, and in particular, major nucleic acid classes, to drive tumor growth, cell motility, angiogenesis, immune evasion and formation of pre-metastatic niches. Circulating sEVs are also utilized as biomarker platforms for diagnosis and prognosis. This review discusses how tumor cells facilitate progression through the metastatic cascade by employing sEV-based communication and evaluates their role as biomarkers and vehicles for drug delivery.

## 1. Tumor Metastasis and Nucleic Acid Cargo in Small Extracellular Vesicles

Cancer is a complex disease, that is driven both by the acquisition of genetic defects and environmental cues. The development of metastatic lesions is a major obstacle impeding curative treatment. The dissemination of cancer cells from a primary lesion to distant organs is referred to as the invasion-metastasis cascade. Due to their systemic nature and the limited treatment options, more than 90% of cancer deaths can be attributed to metastases and not to the primary tumor [1]. The metastatic cascade is a sequence of local and distant events, starting with invasion of primary tumor cells into the blood circulation by the loss of epithelial features, detachment of tumor cells, breakdown of the basement membrane (BM), migration and intravasation. Subsequently, tumor cells need to survive during transport in the circulation, followed by re-attachment and extravasation at the parenchyma of distant tissues [2]. Upon establishment in secondary sites, metastatic cells must adapt and proliferate in a new microenvironment, fostering vascularization to sustain nutrient and oxygen supply during the growth of macroscopic metastases. Each of these events is initiated by the deregulation of signaling cascades due to genetic alterations in cancer cells, but also by signaling from cells in the tumor microenvironment (TME), requiring a coordinated communication between the resident cell types [3]. Besides, tumors were shown to communicate over long distances via the circulation with future metastatic sites to facilitate establishment of suitable pre-metastatic niches (PMNs) [4]. On-going research has attributed a vital part of the coordinated crosstalk in the TME and during the formation of PMNs to small extracellular vesicles (sEVs) and the main, highly abundant sEV-population: exosomes [5,6,7]. sEVs are secreted in large quantities from cancer cells and were originally described as vehicles for cellular waste removal. Further research has indicated that sEVs can also function as vital mediators of intercellular communication by transferring a wide array of bioactive cargos, including proteins, lipids and many different nucleic acids species [8,9]. Two major cargo classes are predominantly involved in the regulation of the metastatic cascade by sEVs: proteins and nucleic acids. In particular, miRNAs and other non-coding RNAs (ncRNAs) were described as the major RNA species in sEVs, but transfer of functional full-length mRNAs was also reported in some instances [10,11]. Intercellular communication utilizing RNA species has been demonstrated during metastasis-associated processes in the TME, such as activation of tumor-associated fibroblasts, tumor cell migration, tumor progression, immunosuppression, angiogenesis and the establishment of organ-specific PMNs [12,13,14,15]. Nucleic acids in sEVs further have promising potential as cancer biomarkers. This includes analysis of sEV-RNAs, but also DNA fragments [16,17,18]. Thus, this review will summarize and discuss the role of sEVs during metastasis in general, including a potential role as diagnostic and prognostic biomarkers, as well as describe the vital contribution of prominent nucleic acid cargos, such as miRNAs during the metastatic cascade.

## 2. sEVs and Their Highly Abundant Exosome-sEV Sub-Population

sEVs are lipid bilayer-engulfed extracellular vesicles with a diameter of 30–150 nm [19]. Based on their size, sEVs are made up mainly by exosomes, but also a sub-population of microvesicles, such as arrestin domain-containing protein 1 (ARRDC1)-mediated microvesicles (ARMMs), with a diameter that reaches below 100 nm, was described [20]. Due to the high abundance of exosomes in the sEV group, this review is mainly focused on the role of exosomes in cancer progression and metastasis. For the sake of an easy communication with the reader, we have however attributed biological effects and in vivo functions to the broader specification “sEVs”, which is often used instead of the term “exosomes” in the literature [21]. The exosome-sEV population is formed as intraluminal vesicles (ILVs) in endosomal-derived multivesicular bodies (MVBs) and released at the plasma membrane. sEVs are present in various body fluids, such as blood, saliva or urine [22]. Omics characterization identified various sEV cargo subtypes, including proteins, lipids and nucleic acids [8,16,17,18,23]. Due to the endosomal origin of respective sEVs, cargo proteins implicated in biogenesis of ILVs, such as tetraspanins (CD9, CD63, CD81, CD82, CD53 and CD37), ALG-2 interacting protein X (ALIX) or the tumor susceptibility gene 101 protein (TSG101), are utilized as markers, e.g., tetraspanins are enriched around 100-fold in sEVs when compared to their parental cells [8,23]. It has to be noted that not all of these proteins are exclusively found in sEVs, and some markers can be detected in microvesicles as well [24]. Moreover, not all tetraspanins are always expressed in a specific cancer cell line. Therefore, the Kalluri group recently performed broad-spectrum mass spectrometry of sEVs across different cancer lines and reported that synthenin-1 was the ideal global marker for sEVs released from cancer cells [25]. sEVs are also enriched in annexins, small Rab-GTPases and lipid-raft-associated factors (e.g., flotillin), as well as major histocompatibility complex class I and II receptors (MHC I and MHC II), heat shock proteins (e.g., Hsp70 and Hsp90), cytoskeleton components (myosin, actin and tubulin), cell membrane receptors, such as integrins, and epidermal growth factor receptor (EGFR), but also cytokines or cytosolic components (e.g., signaling proteins, metabolic enzymes) [26,27]. In addition, sEVs were shown to have a conserved lipid composition that is required for sEV biogenesis, morphology and homeostasis upon uptake. They are enriched in cholesterol, sphingomyelin, glycosphingolipids, phosphatidylserine and ceramide [28,29]. sEVs also contain a large number of nucleic acid classes: mRNAs, miRNAs, long-non-coding RNAs (lncRNAs), circular RNAs (circRNAs), ribosomal RNAs (rRNAs), transfer RNAs (tRNAs) and other RNA subclasses, but DNA was also described [9,26,27]. Thus, sEVs are generated by almost every cell type using multiple biogenesis pathways under physiological and pathophysiological conditions, which vitally determines their respective cargo profiles [30,31].

### 2.1. sEV Biogenesis and Cargo Loading

**Figure 1:**
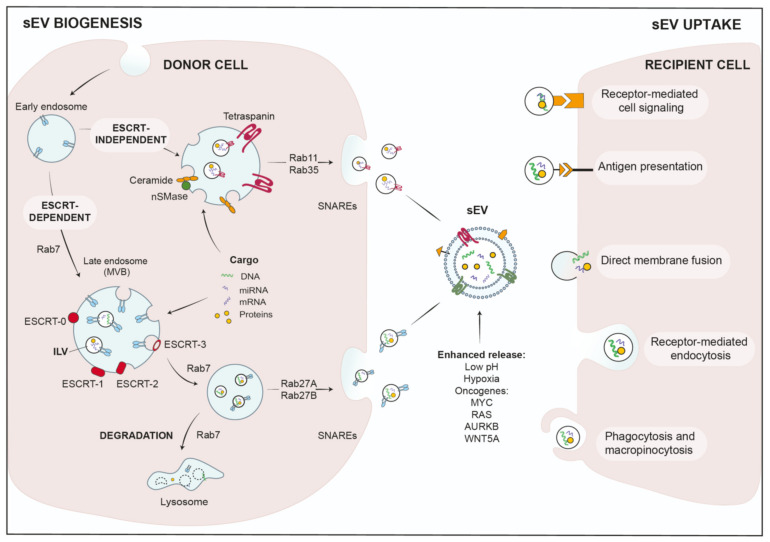
Mechanisms of sEV biogenesis and uptake.

Biogenesis of exosome-sEVs starts at the endosomal compartment by maturing early endosomes into late endosomes or MVBs, where membranes invaginate to generate ILVs [8,32]. Subsequently, MVBs can either fuse with lysosomes and degrade their content, or are transported along microtubules to the cell periphery, where they fuse with the plasma membrane to release ILVs as sEVs [33]. The biogenesis of ILVs can be facilitated by two major pathways: (1) The endosomal sorting complex required for transport (ESCRT) mediates ILV formation by the ESCRT machinery, which is assembled into four larger complexes, ESCRT-0, -I, -II and -III, and aided by the associated proteins AAA-ATPase VPS4, VTA1 and ALIX [34] (Figure 1). To facilitate packaging by the ESCRT complex, cargo proteins need to display post-translational modifications, such as ubiquitination [35]. The ESCRT-0 complex then binds these tagged cargos, segregates the cargo proteins into microdomains and coordinates the additional interaction of ESCRT-1 with these cargos [36,37]. ESCRT-1 in turn recruits ESCRT-2 to facilitate ILV formation and loading of cytosolic proteins or RNAs into ILVs. Subsequently, ESCRT-2 recruits ESCRT-3 to the newly formed vesicles to promote scission of the cargo-laden ILVs together with VPS4, while ubiquitin and ESCRT subunits are released in the cytosol for recycling [36,37,38].

(2) Additional studies have suggested that MVB biogenesis can also work without the ESCRT complex. It was reported that ILVs formed in MVBs even when vital ESCRT subunits were silenced, indicating ESCRT-independent biogenesis [39]. These mechanisms are dependent on tetraspanins and lipids, such as ceramide that is generated by neutral sphingomyelinase 2 (nSMase2). Thus, an inhibitor of nSMase, GW4869, was able to effectively reduce sEV-release in several studies [32,40,41,42]. Mechanistically, lipid-mediated ILV biogenesis is facilitated by spontaneous budding of limited membranes due to incorporation of ceramide, lysophospho- or glycosphingo-lipids. Interestingly, enzymatic conversion of ceramide to sphingosine and sphingosin1-phosphate (S1P), and thus activation of sphingosine1-phosphate receptors on limiting membranes, was also implicated in the sorting of tetraspanins into ILVs [43]. Tetraspanins are major sEV membrane markers, characterized by four transmembrane domains. At the plasma membrane, they are sequestered in tetraspanin-enriched microdomains (TEMs) and interact with a wide variety of associated factors, that can be recycled together with their tetraspanin binding partners into MVBs and eventually ILVs [44] (see Figure 1).

RNA is also loaded in ILVs by a lipid-mediated mechanism. To this end, specific RNA sequences increase the affinity for lipid structures, such as lipid rafts, hydrophobic lipids or sphingosine [45]. In addition, a number of RNA-binding proteins were reported to load mRNAs or miRNAs into sEVs [46]. Concerning miRNAs, Teng et al. demonstrated removal of the tumor-suppressor miR-193 from cells during colon cancer progression by packaging in sEVs, utilizing the miRNA-binding protein, major vault protein (MVP) [47]. Thus, sEVs are used by cancer cells in order to rebalance their content of tumor-suppressive and oncogenic miRNA populations [47].

Upon completion of ILV biogenesis, MVBs are transported along microtubules to the plasma membrane, where ILVs are released [48,49]. Here, several factors are involved on the way, which include small Rab family GTPases as molecular switches, microtubules and their associated regulatory proteins, molecular motors that transport the MVBs and soluble N-ethylmaleimide-sensitive factor attachment protein receptors (SNAREs) that mediate final membrane fusion. In particular, the Rab-GTPases are vital regulators of vesicular trafficking and ILV budding or release, e.g., Rab27a/b or Rab11 regulate different aspects of ESCRT-dependent or independent sEV-release [9,50,51] (Figure 1). The efficient fusion and sEV-release at the plasma membrane also requires the presence of branched actin filaments. Branched actin is either stabilized or debranched by the actin-regulatory proteins Cortactin and Coronin-1, respectively [52]. Cortactin also facilitates Arp2/3-complex-dependent synergistic nucleation of branched actin filaments together with nucleation-promoting factors [53]. Our group has recently shown that Cortactin together with WAVE2 is required for sEV-release from pancreatic ductal adenocarcinoma (PDAC) cells. In this study, we have also described Protein Kinase D1 (PRKD1) as a vital inhibitory upstream regulator, that phosphorylates Cortactin at S298 [54,55]. When phosphorylation of this site was abrogated, Cortactin induced synergistic nucleation of branched filaments required for sEV-release, thus drastically increasing the respective sEV-secretion [54]. Interestingly, PRKD1 expression is reduced in a majority of PDACs and other invasive cancer cells [54,56]. In summary, sEV biogenesis can occur in an ESCRT-dependent and independent manner in a highly regulated process that involves the coordinated action of multiple cellular compartments as well as tight spatio-temporal control.

### 2.2. sEV-Uptake in Recipient Cells

Once released into the extracellular milieu or the circulation, sEVs can interact with recipient cells by different mechanisms. They can either bind to membrane receptors and activate specific signaling pathways, or cargo is transferred upon uptake into the respective cells. Mechanisms for uptake and delivery of active biomolecules include direct membrane fusion, clathrin- or lipid raft (Caveolae/caveolin-1)-mediated endocytosis, macro-pinocytosis and phagocytosis [57] (see Figure 1). In addition, molecular and cellular stress conditions were shown to modify sEV-release and -uptake rates, as well as their respective cargo composition [9].

### 2.3. Modifiers of sEV-Release in Tumor Cells

Tumor cells are associated with strongly increased sEV-release [58,59,60,61], which further display a significantly altered cargo profile to act as signaling hubs for intercellular communication during tumor metastasis. Thus, quantitative and qualitative changes in sEV populations were identified in the blood circulation of cancer patients, corroborating a function of sEVs as diagnostic and prognostic markers [62,63,64,65]. The molecular mechanisms involved in sEV trafficking and release are complex and not fully understood. However, it has become evident that cellular and molecular stresses, such as environmental cues and oncogenic transformation, have a crucial role in triggering sEV secretion [66,67]. One hallmark of the TME is low pH, which was shown to be a key factor for sEV-release, but also sEV-uptake in recipient cells [66]. Similar observations were made for hypoxic conditions, where the low oxygen concentrations not only quantitatively alter sEV-release, but also facilitate qualitative changes in the sEV cargo content to promote vascularization and cell proliferation [67]. Since ESCRT-dependent and independent pathways are major regulators of sEV biogenesis, interference with the respective pathways was able to potently modulate secretion [68,69,70,71]. A recent study has demonstrated that oncogenes found in many cancer entities, such as MYC, aurora kinase B (AURKB) and HRAS, trigger hyperactivation of ESCRT and ceramide pathways, as well as the inhibition of lysosome genes causing abundant sEV secretion. Again, the oncogenes mediated a shift in cargo composition, in particular for proteins and miRNA, thus promoting a pro-tumorigenic phenotype [71]. In another setting, oncogenic HRAS induced considerable release of sEVs from epithelial cell lines, which were shown to contain the whole cancer cell genome, including the mutant HRAS oncogene [72,73]. Moreover, several lines of evidence suggest that the KRAS oncogene was able to enhance sEV-release and modulate their functional cargo compared to wild-type KRAS. Mutated, oncogenic KRAS-sEVs were characterized by tumor-promoting proteins, including mutant KRAS, and an altered miRNA content, enabling oncogenic transfer and metabolic reprograming in recipient cells [74,75,76]. Different mechanisms are thought to be involved in RAS-dependent regulation of sEV-release, e.g., the activation of syndecan-1, as well as the RHO pathway, which have both functions in sEV biogenesis [77,78,79]. In addition, the Wnt protein WNT5A, an oncogene correlating with metastasis and poor prognosis, was able to trigger Ca2+-dependent release of sEVs with pro-angiogenic and immunosuppressive features [80]. There are opposing observations and hypotheses regarding the miRNA content of oncogene-induced sEVs. Several studies report the release of pro-tumor miRNAs through sEVs by cancer cells to reprogram recipient malignant and non-malignant cells, enabling cancer progression [81]. On the other hand, oncogene-induced sEVs were found to be enriched in miRNAs with tumor-suppressor functions, supporting the hypotheses that tumor cells eliminate undesirable cellular miRNAs as cargo in these sEVs [71,75].

Since nucleic acids and in particular miRNAs have vital functions during tumor progression and metastasis, we were prompted to focus this review on delineating the contribution of sEV-based nucleic acid transfer during progression through the metastatic cascade.

## 3. Role of sEVs in Local Stroma Invasion

The first steps of cancer metastasis are dictated by the local invasion from the primary tumor site into the surrounding stroma. Tumor cells need to breach the BM, the extracellular matrix (ECM) which separates epithelial from stromal compartments and acts as a barrier to invasiveness [82]. The detachment from the BM is initialized by the loss of cell–ECM and cell–cell adhesive contacts, such as different adherens and tight junctions that maintain cellular connections, e.g., by E-cadherin and claudins, which under normal conditions facilitate the formation of a tight epithelium. During cancer progression and invasion, these structures are weakened or disassembled, as a consequence of mutations and dysregulated signaling in order to allow the invasion into the surrounding stroma [83]. A process implicated in the disassembly of epithelial cell–cell interactions and acquisition of invasive features is epithelial-to-mesenchymal transition (EMT) [84].

### 3.1. Epithelial-to-Mesenchymal Transition (EMT)

During EMT, cells lose epithelial traits and transform into a motile, mesenchymal state. EMT can be observed during embryonic development and wound-healing processes, where the loss of adhesion properties, polarity and basement anchoring are important physiological events. However, when cancer cells activate the EMT program, they gain stemness and migratory abilities, which contribute to invasiveness and ultimately metastasis [85]. On a molecular level, EMT includes loss of epithelial markers, such as E-cadherin, and upregulation of mesenchymal proteins, e.g., vimentin, fibronectin, N-cadherin, as well as the change to a fibroblast-like cell [3]. The downregulation of E-cadherin and upregulation of mesenchymal markers during EMT is orchestrated by zinc-finger transcription factors, such as SNAIL, SLUG, TWIST and ZEB-1/2, downstream of growth factor signaling induced by hepatocyte growth factor (HGF), epidermal growth factor (EGF) and transforming growth factor beta (TGFβ), or Wnt/β-catenin pathways [84,86,87,88]. However, the transcriptional deregulation and induction of EMT is also prominently facilitated by uptake of sEVs, which modulate signaling of the respective pathways, e.g., upon transfer of mir-301a in sEVs, targeting p63 and thus release/activation of ZEB1/2 [89]. EMT is also promoted by the transfer of constitutively active β-catenin or Wnt ligands (Wnt1 and Wnt3a), as well as sEV-resident miRNAs mir-92a [90], miR-191 [14] and miR-1260b [91], implicated in modulating Wnt/β-catenin signaling. However, loss of epithelial characteristics and detachment from the ECM also pose a risk for cancer cells by triggering anoikis, a form of programmed cell death to prevent metastasis. Thus, cancer cells need to acquire anoikis resistance, which can occur via several mechanisms, such as the transfer of sEVs containing miRNA-210 and miR-222-3p, as shown for non-small-cell lung cancer (NSCLC) and gastric cancer cells (GC) [92,93].

### 3.2. ECM Degradation

The invasion of cancer cells is further supported by the degradation of the BM and other ECM structures in the tissue. Key players in ECM remodeling are matrix metalloproteinases (MMPs), whose activity is controlled by transcriptional and post-translational regulation. Cancer cells have acquired different mechanisms to deactivate this tight control of MMPs and render them highly active. Enhanced function of several MMPs was shown to regulate ECM stiffness via integrins and contributed to invasive phenotypes of cancer cells [94]. Moreover, MMP expression was shown to release growth factors, such as TGFβ, from the TME by influencing their bioavailability or functionality, thus enabling cell proliferation [95,96]. Interestingly, a study by Yokoi and colleagues in 2017 established that sEVs may play a crucial role in fostering MMP activity. Here, no MMP protein was found in sEVs, as shown in various studies [97,98,99], but the selective packaging of MMP1 mRNA into sEVs derived from ovarian cancer (OC) cells was found. These sEVs then triggered destruction of the peritoneal mesothelium and BM in vitro and in vivo, ultimately promoting metastatic behavior [100]. Several studies have found miR-21 to be overexpressed in different cancer types and demonstrated its transfer via sEVs to recipient cells [101,102,103]. miR-21 positively correlated with the expression of ECM mediators MMP-2, MMP-9 and MMP-11, due to the potent inhibition of phosphatase and tensin homolog (PTEN) and inhibitor of metalloproteinases 3 (TIMP-3) [102,104]. Similar effects have been established for miR-181b during carcinogenesis in hepatocellular carcinomas (HCC) and esophageal cancer (ESCC) [105,106].

## 4. sEV-Based Communication in the Tumor Microenvironment (TME)

Once cancer cells have breached the BM, they invade the stroma, where they are exposed to stromal cells. These non-malignant cells comprise fibroblasts, myofibroblasts, vascular and lymphatic endothelial cells, adipocytes and infiltrating immune cells [107]. The immediate ECM and cellular components of the stroma make up the TME. The classical theory suggests that upon cancer initiation, adjacent non-transformed stromal cells are recruited and reprogrammed, which is accompanied by extensive intercellular communication via cytokines, chemokines and vesicles to generate a dedicated favorable TME [108]. Chronic inflammation and wound-healing processes in an aberrant microenvironment also promote tumorigenesis [109,110,111]. Research in recent years has reinforced the crucial role of crosstalk between cancer cells and cellular components of their TME to create a supportive environment, thus enhancing the aggressive and invasive behaviors of tumors. Reprogrammed stroma cells enable the supply with essential nutrients in order to meet the high demand of proliferating tumor cells, but also to remodel the ECM and escape immune surveillance [107]. This communication is largely dependent on soluble factors and the transfer of sEVs. Tumor-sEVs or sEV-based crosstalk have been described for many cell types in the TME, such as cancer-associated fibroblasts (CAFs), which facilitate cancer progression either in an autocrine or paracrine fashion, e.g., by inducing EMT or stemness [112]. Infiltrating innate and adaptive immune cells, such as tumor-associated macrophages or T_regulatory_ cells (T_reg_), also generate an immunosuppressive TME [113]. Moreover, tumor angiogenesis is induced by tumor-derived-sEVs [114,115]. Once sEVs have entered the circulation, they were recently also described to foster metastasis in distant organs by establishing favorable PMNs [5,54,116].

### 4.1. sEV-Based Crosstalk with Cancer-Associated Fibroblasts (CAFs)

CAFs are the most common constituent of the TME. Their crosstalk with tumor cells and the extended tumor stroma influences invasion, metastasis as well as therapeutic responses. When normal fibroblasts (NFs) are converted into CAFs, they acquire the expression of specific protein markers, including fibroblast activation protein (FAP), alpha-smooth muscle actin (α-SMA) and fibronectin. Activated CAFs in turn secrete growth factors, e.g., vascular endothelial growth factor (VEGF), TGFβ, cytokines (IL6, IL10, IL1β), collagen and ECM-modifying enzymes, but also sEVs. These secreted mediators then act on tumor cells in a paracrine manner to alter the ECM and further facilitate tumor invasion across tissues [117]. sEVs have shown the ability to act on the TME and convert NFs into CAFs. A mechanism involved in cellular differentiation of fibroblasts to CAFs by sEVs is activation of the TGF-β/Smad pathway. This is either accomplished by direct transfer of growth factors on sEVs or by transcriptional reprogramming induced by the introduction of ncRNAs. In prostate cancer (PC) cells, the direct delivery of TGFβ to fibroblasts by sEVs initiated a phenotype resembling stromal cells from cancerous prostate tissue and the elimination of sEVs in vivo abolished CAF formation [6]. Similar observations were made for breast cancer (BC) [118], OC [119] and GC [120]. Additional examples for a role of sEVs in CAF conversion include melanosomes, sEVs derived from melanocytes, containing mir-211, which targets the tumor suppressor insulin-like growth factor 2 receptor in normal fibroblasts, resulting in the activation of mitogen-activated protein kinase (MAPK), thus enabling CAF reprogramming [12]. MiR-9 is upregulated in several BC cell lines and can be transferred via sEVs to recipient breast fibroblasts, thereby inducing CAF-like properties [121], whereas in PDAC, miR-155 was shown to activate NFs [122]. Once CAFs are transformed and activated in the TME, they also secrete a plethora of functional non-tumor sEVs with altered ncRNA composition and the ability to promote cancer progression as well as metastasis (Table 1). For example, sEVs released from CAFs were shown to regulate BC cell migration and invasion by transfer of mir-181d-5p, targeting CDC2 and HOXA5 to enhance proliferation, EMT and aggressiveness [123]. In addition to CAFs, the TME is also characterized by the infiltration of immunosuppressive cells, which help the tumors to avoid immune surveillance.

### 4.2. sEVs in Immune Suppression

Tumor-derived sEVs are targeted towards different innate and adaptive immune cells in the TME or at metastatic sites to facilitate immune evasion. Usually, antitumor immunity is triggered by the release of tumor-associated antigens (TAAs) and the subsequent activation of innate and adaptive effector cells, e.g., natural killer cells (NKs) and CD8+ T_effector_ cells. In particular, the activation of CD8+ T_effector_ or CD4+ T_helper_ cells was shown to be suppressed by tumor-derived sEVs, e.g., upon targeting antigen-presenting dendritic cells (DC), thus impairing lymphocyte activation and survival [162].

In immunologically cold tumors, such as PDAC, the TME is also spiked with a large number of immunosuppressive regulatory T-cells (T_regs_), M2-polarized tumor-associated macrophages (TAMs) and immature myeloid-derived suppressor cells (iMDSCs), which inhibit functional CD8+ T-cell responses and impede proper antigen presentation by DCs or anti-tumor responses by M1-polarized macrophages [163].

Besides, sEVs secreted by tumor cells often reflect the parental protein composition, and thus were shown to present TAAs on MHC class I and II receptors. These sEV-based TAAs are able to stimulate NK- and T-cell-dependent cytotoxicity, but were further shown to decoy anti-TAA-antibodies and thus prevent complement-mediated cytotoxicity, impairing functional anti-tumor B-cell responses [164].

To escape immune surveillance, tumors have acquired diverse mechanisms that target the innate and adaptive immune defense by sEVs. The respective concepts, targeted cells and the role of nucleic acids are summarized in the following section. A more extensive overview is available in Table 1.

#### 4.2.1. Innate Antitumor Defense—Natural Killer Cells (NKs)

NKs mediate the innate defense against tumors or infected cells [165]. The antitumor cytotoxicity of NKs can be blocked by inhibiting the activating NK cell surface receptor NK group 2-member D (NKG2D). To this end, membrane-bound ligands MICA, MICB and ULBP against NKG2D are transferred by sEVs, thus alleviating NK-mediated cytotoxic responses [166,167]. Hypoxic tumor MVs, comprising of tumor sEVs and microvesicles (MVs), also impaired NK cell cytotoxicity against different tumor cells in vitro and in vivo. This was mediated by EV-resident TGF-β1, decreasing the expression of NKG2D, while miR-23a synergistically targeted CD107a expression in NK cells. Another mechanism to impair NK cell function is the sEV-mediated transfer of the circular RNA:ubiquitin-like PHD and ring finger domain 1 (circUHRF1), which was associated with the suppression of miR-449c-5p and enhanced expression of TIM-3. Interestingly, in HCC patients, increased levels of plasma-circUHRF1 were also correlated with resistance to anti-PD-1 therapy [131]. The cytotoxicity of NK cells was further inhibited by tumor sEVs from different cancer cell lines [133] by suppressing the secretion of granzyme-B via miR-378a-3p.

#### 4.2.2. M2-Polarized Macrophages

In addition to NKs, tumor sEVs facilitate the transformation (polarization) of TAMs into immunosuppressive M2 subtypes, e.g., sEVs derived from hypoxic PDAC cells convert macrophages to the M2 phenotype in a HIF1a- or HIF2a-dependent manner. miR-301a-3p is highly expressed in the respective cells as well as sEVs under hypoxic conditions. Upon uptake by macrophages, M2-polarization is induced via the activation of PTEN/PI3Kγ signaling. The respective macrophages in turn also promote tumor-invasive behavior. The circulating exosomal miR-301a-3p levels were thus positively associated with depth of invasion, lymph node metastasis, late TNM stage and poor prognosis in patients [132].

#### 4.2.3. Adaptive Antitumor Immunity—Targeting Antigen-Presenting Dendritic Cells (DCs) and Anti-Tumor T-Cells

Tumors can also bypass surveillance by the adaptive immune system utilizing sEVs. Major targets include antigen-presenting DCs, CD8+ T_effector_ and CD4+ T_helper_ cell types, as well as regulatory T_regs_, which are immunosuppressive and promote tumor growth and metastasis. For example, in PDAC, T-cell activation by antigen-presenting cells was impaired upon uptake of PDAC-derived sEVs in DCs, e.g., uptake of miR-203-containing PDAC-sEVs inhibited the expression of toll-like receptor 4 (TLR4), tumor necrosis factor α (TNF-α) and interleukin IL-12, inducing DC dysfunction [136,168]. PDAC-sEVs can also specifically impact the activation of CD4+ T-cells by DCs. This was mediated by inhibiting the expression of the regulatory factor X-associated protein (RFXAP) with miR-212-3p, resulting in compromised MHCII expression. Such a phenotype could also be validated in the tissue of PDAC patients [134]. In addition to tumor cells, T_regs_ possess the ability to produce sEVs that induce DC tolerogenic phenotypes. Mechanistically, this was attributed to the sEV miRNA cargos miR-150-5p and miR-142-3p, that are transferred with T_reg_-derived sEVs, which trigger IL-10 and IL-6 production in tolerogenic DCs to limit T-cell responses [135].

Additionally, the different T-cell populations are impacted by various sEVs-dependent mechanisms. This includes induction of apoptosis, as well as inhibition of CD8+/CD4+ T-cell activation, expansion and conversion towards immunosuppressive T_reg_ phenotypes [169]. Apoptosis in CD8+ T-cells is mediated by different proteins and nucleic acid cargos, e.g., the interaction between Fas ligand (FasL) on sEVs and Fas receptors (CD95) on the respective T-cell clones, resulting in caspase activation [170]. The presence of FasL on sEVs was reported for different cancer entities, such as melanoma [171], PC [172] as well as head and neck cancer [173]. In melanoma, tumor sEVs also induce the mitochondrial apoptotic pathway in CD4+ T-cells, and this is mediated by transfer of miRNA cargos miR-690, miR-677 as well as miR-29b [130]. Another mechanism of immune evasion involves binding of programmed death-ligand 1 (PD-L1) expressed on tumor cells and other cells in the TME to its receptor programmed cell death protein 1 (PD-1) on activated T-cells, thus triggering T-cell exhaustion by suppressing activation and expansion [174,175]. Interestingly, tumor-derived sEVs were also described to transfer PD-L1 to other cell populations in the TME, thereby amplifying immunosuppression. Metastatic melanomas release PD-L1-positive sEVs, which enable tumor growth due to the suppression of CD8+ T-cell cytotoxicity. The level of PD-L1 in circulating sEVs could even be utilized for the stratification of early-stage responders and non-responders after anti-PD-1 therapy [176]. Release of PD-L1-positive sEVs has been observed for BC [177], NSCLC [178], glioblastoma (GBM) [179], GC [180] as well as other cancer entities. In cervical cancer (CeC), increased PD-L1 levels can also be traced back to the upregulation of miR-18a, which targets PTEN and SOX6 [137], whereas in HCC, miR-23a-3p induced elevated PD-L1 levels in macrophages [138]. For both miR-18a and miR-23a-3p, transfer by tumor sEVs has been reported [139,181]. sEVs-based signaling further interfered with T-cell polarization and cytokine release. Here, sEV-resident miRNAs, hsa-miR-24-3p, hsa-miR-891a, hsa-miR-106a-5p, hsa-miR-20a-5p and hsa-miR-1908, are upregulated in the serum of patients with nasopharyngeal carcinomas. The respective sEVs impeded T-cell proliferation and Th1/Th17 differentiation in vitro, accompanied by decreased levels of IFNγ, IL-2 and IL-17, while on the other hand, immunosuppressive T_regs_ were promoted. This was facilitated by altered mitogen-activated protein kinase (MAPK) and STAT signaling [129].

#### 4.2.4. Myeloid-Derived Suppressor Cells (MDSCs)

MDSCs are immature myeloid cells with immunosuppressive features that are present in the TME of many tumors. MDSCs decrease the cytotoxicity of effector immune cells and increase T_reg_ cell responses, thus contributing to cancer progression. In several cancer subtypes, circulating MDSC levels were shown to correlate with the clinical stage and therapeutic response in patients [182].

A study by Guo and colleagues established a role for glioma cell-derived sEVs in potentiating the activation of MDSCs, targeting RAR-related orphan receptor alpha (RORα) and PTEN with miR-10a and miR-21. These findings were further corroborated by inoculation experiments in mice, where glioma cells with miR-10a or miR-21 knockout generated a lower number of MDSCs than normal glioma cells [140]. In PDAC, loss of the tumor suppressor SMAD4 was associated with poor prognosis as well as production of sEVs containing miR-1260a and miR-494-3p. The respective sEVs were reported to reprogram granulocytic and monocytic g/mMDSCs to bolster proliferation and glycolysis, thus facilitating the establishment of an immunosuppressive TME [183].

In summary, tumor sEVs and nucleic acid cargos have vital functions in evading immunosurveillance by the innate and adaptive immune system, fostering tumor growth and progression towards metastasis.

### 4.3. sEVs in Angiogenesis

As tumors grow and disseminate, they are critically dependent on access to blood vessels. Angiogenesis is a multi-step process initiated during carcinogenesis to form new blood vessels from pre-existing ones. Angiogenesis facilitates the adequate supply of oxygen and nutrients, as well as the removal of waste products from cancer cells, and is therefore essential for tumor proliferation and metastasis [184]. Hypoxic and acidic conditions, encountered in the TME of most tumors, promote new vascularization due to the release of pro-angiogenic factors, such as VEGF [185]. Tumor-derived sEVs are further implicated in pro-angiogenetic signaling, e.g., sEVs from GBM stem-like cells were shown to transport VEGF-A to brain endothelial cells in order to activate the VEGF pathway, initiating blood vessel growth and enhanced vascular permeability [114]. The presence of VEGF has also been reported in melanoma-derived sEVs, thus enhancing the angiogenic capacity of respective tumors [80]. Besides VEGF, tumor-derived sEVs transfer many more proteins, e.g., MMPs [186], involved in blood vessels’ formation. Moreover, several miRNAs present in tumor-derived sEVs were found to prominently facilitate angiogenesis in endothelial cells (Table 1). miR-17-5p is loaded in sEVs of nasopharyngeal carcinoma cells suppressing the transmembrane protein BMP and activin membrane-bound inhibitor (BAMBI). As a consequence, expression of AKT/VEGF-A and thus angiogenetic activity of HUVEC cells was increased. A positive correlation between miR-17-5p in the serum of nasopharyngeal carcinoma patients and tumor angiogenic activity further demonstrated the importance of this miRNA in vivo [115]. In GC, sEV-resident miR-155 was show to target Forkhead box O3 (FOXO3a) as well as c-MYB, thus enhancing the expression of VEGF in vascular cells to induce angiogenesis and tumor growth [150]. In PDAC, mir-27a in sEVs induced vascularization through inhibition of BTG anti-proliferation factor 2 (BTG2) [13]. Interestingly, sEVs secreted under hypoxic conditions showed the greatest potential to promote blood vessel formation, when compared to normoxic sEVs due to their enrichment of pro-angiogenic miRNAs, such as miR-135b, miR-210, miR-21 and miR-23a [151,152,153,187]. In addition to miRNAs, other ncRNAs are also packed in sEVs to foster tumor angiogenesis. In OC, high expression of the lncRNA MALAT1 (metastasis-associated lung adenocarcinoma transcript-1) was correlated with angiogenesis and metastasis, and MALAT1 was found in sEVs, which induce pro-angiogenic gene expression upon uptake in HUVEC cells [154].

Additionally, lymphatic vessels play crucial roles in tissue homeostasis, fluid balance, immune function as well as transport of metabolic molecules, and are therefore important for tumor proliferation [188]. Furthermore, they lack the presence of a complete BM and are more permeable to tumor cells, thus fostering metastasis [189]. Tumor cells have been shown to secrete growth factors such as VEGF-C or FGF to induce the formation of new lymphatic vessels (lymph-angiogenesis), correlating with lymph node as well as distant metastasis [190,191,192]. Besides, tumor-derived ncRNAs were shown to influence lymphatic vessel growth. miR-221-3p packaged into sEVs of CeC was identified to promote expansion of lymphatic vessels by targeting vasohibin-1 (VASH-1), a negative regulator of lymph-angiogenesis [156]. Additionally, the lncRNA lymph node metastasis-associated transcript 2 (LNMAT2) in sEVs secreted from bladder cancer was reported to facilitate lymph-angiogenesis via VEGF-C-independent mechanisms [157].

## 5. Intravasation of Tumor Cells and Survival in the Circulation

One of the most crucial steps enabling metastasis is the intravasation of tumor cells into the blood circulation. Neo-angiogenesis and lymph-angiogenesis induced by tumors, as well as tumor-derived sEVs, favor this process, since the newly developed vasculature is more prone to leakiness [193]. Thus, a weak interaction between endothelial cells facilitates transmigration of cancer cells through the endothelial monolayer into the circulation. Additionally, intravasation is promoted by TGFβ, enhancing penetration of tumor cells through microvessel walls [194], as well as TAMs, which foster paracrine interactions with tumor cells and an altered microvessel density [195]. Moreover, sEV-derived miRNAs, which disrupt endothelial stability, promote the process of tumor cell intravasation (Table 1). For example, miR-939 transferred by BC-sEVs targeted vascular endothelial (VE) cadherin, the main constituent of adherens junctions, causing enhanced leakiness of blood vessels, whereas miR-105 downregulated the tight junction protein ZO-1 [147,148]. Upon their successful intravasation, cancer cells can disseminate widely in the blood stream as circulating tumor cells (CTCs) and travel to distant sites, where they seed metastatic lesions. However, CTCs must survive in the circulation, and this is challenged by a variety of stress conditions, such as risk of anoikis in the absence of anchorage, shear forces and immunologic surveillance [196]. CTCs can be detected as single cells or dense clusters from 2 up to >100 cells with strong cell–cell junctions [107]. The aggregation of CTCs is of advantage, since clustered cells are protected from shear stress and anoikis. Thus, the presence of CTC clusters is associated with a poor prognosis and an earlier onset of metastasis [197,198,199]. CTC clusters directly derive from the primary tumor or develop via intravascular aggregation of single CTCs [200,201]. In HCC, tumor-derived sEVs were shown to transfer SMAD Family Member 3 (SMAD3) protein and mRNA to detached HCC cells and promote their homotypic adhesion [202]. Furthermore, sEVs have the ability to indirectly facilitate CTC clustering via the regulation of adhesion proteins, as well as the conversion of fibroblasts into CAFs, which also play a major role in cluster formation [203,204,205,206]. Furthermore, CTCs directly interact with blood platelets, which then accompany cancer cells and protect them from recognition and lysis by NKs [207,208]. The importance of platelets was further corroborated, since their depletion inhibited the formation of metastasis in mice [209]. This phenomenon can also lead to an imbalance in the normal blood clotting, which is often observed in cancer patients with a poor prognosis [210,211,212]. Thrombotic events and the activation of platelets in cancer patients are strongly correlated with the overexpression of tissue factor (TF), which can be transferred to endothelial cells via tumor-derived sEVs. The ECs are then reprogrammed into a procoagulant phenotype and generate high levels of thrombin, one of the most important activators of platelets [213]. In turn, platelets secrete sEVs with pro-metastatic cargoes, such as TGFβ, integrins, P-selectin and glycoprotein IIb–IIIa, that reprogram endothelial cells, leukocytes and tumor cells [214,215,216]. Moreover, the aggregation of platelets and inhibition of coagulation inhibitors are triggered by the formation of neutrophil extracellular traps (NETs), that can also be induced by tumor-derived sEVs [217].

At the moment, the influence of sEV-derived miRNAs during the clustering of CTCs as well as the activation of platelets and NETs is unclear, and further research is needed to unravel potential mechanisms. However, several miRNAs contained in sEVs play a crucial role in protecting CTCs in the blood stream from being recognized by the immune surveillance due to suppression of immune cells or recruitment of T_regs_, which is reviewed as part of Section 4.2.

## 6. Extravasation from the Circulation

Extravasation occurs when CTCs transmigrate through the endothelial wall in order to enter the parenchyma of distant tissues. This process is dependent on enhanced vascular permeability and the disruption of cell–cell junctions, which normally maintain a physical barrier for fluid proteins and cells. Several secreted factors from cancer cells are involved in the disruption of endothelia barriers, e.g., TGFβ, angiopoetin-like 4 (ANGPTL4), VEGF, MMPs and ADAM12 [218,219,220]. Additionally, the association of platelet-cancer cell hybrids to the vessel wall is a step in the extravasation of CTCs, and this is guided by interactions of platelet selectins with ECs [221]. As described for intravasation, several miRNAs in sEVs support transendothelial migration by weakening endothelial cell–cell junctions, thus enabling vascular permeability (Table 1). However, the question arises if extravasation occurs just randomly or if CTCs are specifically directed towards distinct organs niches, facilitating organotropic metastasis.

## 7. sEV-Mediated Organotropism and Formation of Pre-Metastatic Niches

The concept of organotropism describes the homing of circulating tumor cells to specific organs as a consequence of complex tumor–stroma interactions. Although organotropic metastasis was first hypothesized 1889 by Stephen Paget, the exact underlying mechanism is not yet fully unraveled. Recent studies have indicated that sEVs may play a crucial role in organotropism. sEVs prepare PMNs by recruiting bone marrow-derived cells (BMDCs), endothelial progenitor cells and mesenchymal cells, but also induce the upregulation of proinflammatory molecules and facilitate vascular leakiness to create a suitable niche environment [222,223]. These alterations in distant organs are already observed before the arrival of cancer cells. Interestingly, various studies indicate that sEVs from different types of tumors preferably migrate to distinct organs, e.g., melanoma sEVs to sentinel lymph nodes, BC-sEVs to the lung and PDAC-sEVs to the liver [224,225,226]. Thus, the question arises of how sEVs are directed to specific sites to enable organotropic metastasis. In 2015, Hoshino et al. established that the expression of integrin patterns in sEVs and their interaction with ECM molecules, such as laminin and fibronectin, is crucially important for governing the formation of organ-specific PMNs. Integrins α6β4 and α6β1 were shown to promote lung tropism, and their uptake by lung fibroblasts triggered expression of the pro-inflammatory S100A proteins, heat shock proteins (HSPs), laminin and fibronectin, as well as annexin A6 and CD44 [5,227,228,229]. sEVs positive for integrins avß5 and macrophage migration-inhibitory factor (MIF) favor liver organotropism, a common site for metastasis associated with a poor prognosis in many types of cancer [226]. These sEVs bind to the fibronectin-enriched ECM in the liver and educate liver-resident macrophages (Kupffer cells) to increase the expression of TGFβ. High TGFβ levels in turn activate the secretion of fibronectin and pro-inflammatory mediators from hepatic stellate cells, thus preparing the PMN for the tumors [5,226].

Our group has recently observed that in PDAC, PRKD1 expression is downregulated compared to non-tumor tissue. Impaired PRKD1 expression was associated with increased sEVs secretion from different PDAC cell lines as well as altered expression and loading of sEVs with integrin α6β4. The respective sEVs also displayed low levels of integrin β5, impairing the formation of functional αvβ5 dimers, due to transcriptional downregulation of integrin β5 expression upon PRKD1 loss. In line with the model of Hoshino et al., injection of PRKD1^KO^-sEVs from Panc-1 cells in subcutaneously xenografted mice promoted lung metastasis, and this was corroborated in an autochthonous Prkd1^KO^KC mouse model, were predominant lung and no macroscopic liver metastasis was detected [54].

Interestingly, another mechanism for sEV-mediated liver metastasis was recently established by Zhang et al. Here, the transfer of EGFR by GC-sEVs into the liver facilitated HGF activation via miR-26a/b suppression. HGF was shown to bind c-MET on cancer cells and thus rendered the liver a preferable metastatic site for multiple tumors [230].

The establishment of bone metastasis can be observed for many solid tumors and often occurs in advanced disease stages. In bone metastasis, the homeostatic balance between bone-producing osteoblasts and bone-resorbing osteoclasts is disrupted and metastatic lesions are either osteolytic or osteoblastic. There are various studies assigning a crucial role for sEVs in inducing said imbalance, e.g., via transfer of amphiregulin, causing osteoclastogenesis through upregulation of receptor-activator of NF-κB ligand (RANKL), a mechanism that is observed in NSCLC [231].

Although most studies have defined a crucial role for integrins and other protein cargos in the establishment of organ-specific PMNs, a few miRNAs are also known to contribute to organotropic metastasis. One important mediator is miR-141-3p, and its presence in sEVs was associated with metastatic PC, promoting osteoblast activity and bone metastasis [15]. Similarly, miR-940 from PC and BC cells was reported to facilitate osteoblastic lesions by targeting Rho-GTPase-activating protein 1 (ARHGAP1) and family with sequence-similarity 134, member A (FAM134A) [158]. In the PMN, sEV-derived miRNAs further reprogram non-tumor stromal cells, promoting a pro-tumor environment. Thus, alterations in glucose metabolism are frequently observed in the PMN in order to fuel nutrient demand of incoming cancer cells. Here, miR-122 from BC-sEVs was described to target and downregulate pyruvate kinase, in order to impair glucose uptake in lung- and brain-resident cell populations to supply cancer cells with the remaining glucose [116]. Similar to the primary tumor site, the sEV-induced conversion of fibroblasts into CAFs is also observed in the PMN. Relevant nucleic acid targets are listed in Table 1. One specifically important cargo for the lung PMN is miR-1247-3p, that is secreted in sEVs from highly metastatic HCC and induces the activation of NF due to the upregulation of NFκB signaling, enabling the release of pro-inflammatory cytokines to further niche formation [159].

In summary, PMN formation has emerged as a vital step during metastasis and is crucially determined by tumor-derived sEVs. Although most of the important sEV cargos involved in the regulation of niche establishment are proteins, some miRNAs have been described to facilitate or aid niche formation, and thus more research is needed to unlock the full potential of nucleic acids in the regulation of organotropic metastasis.

## 8. sEVs as Biomarker Platforms and Therapeutic Vehicles

Since sEVs are representative for their cells of origin and are released in large quantities by cancer cells in the bloodstream [22,64], they are ideal biomarker platforms for liquid biopsy to facilitate early detection and prognosis [62,63]. Different sEV cargo classes can be utilized as promising prognostic and diagnostic biomarkers upon liquid biopsy, e.g., proteins, lipids and nucleic acids are intensively investigated. For example, glypican-1 was identified as a promising diagnostic protein biomarker for PDAC [232], but the ratio of lipids in plasma or serum sEVs was also used for cancer diagnosis and prognosis (reviewed in [233]). To this end, specific ratios of lysophosphatidylcholine (LPC), phosphatidylcholine (PC) and phosphatidylethanolamine (PE) in sEVs were associated with the tumor stage of PDAC, and PE was even linked to overall patient survival [234]. However, this review mainly focuses on the role of nucleic acids, and in particular ncRNAs in sEVs, as markers for diagnosis and prognosis in different cancer entities. A major obstacle for the use of sEVs as reliable biomarkers in the clinical routine is reproducibility and specificity due to different isolation and detection methods. sEV isolation for biomarker studies so far is not standardized, and each method, such as ultracentrifugation, sucrose gradients, size exclusion chromatography, affinity-based purification, isolation by asymmetric flow field–flow fractionation or microfluidic devices, has its own advantages and downsides [235,236,237]. This has become evident in the most studied sEV marker for PDAC diagnosis, glypican-1. Glypican-1 was discovered by Melo et al. in animal and human cell lines and was described to demonstrate a sensitivity/specificity of 100%, upon detection by transmission electron microscopy on sEVs. Detection of glypican-1-positive sEVs by ELISA reduced the sensitivity and specificity to 82.14% and 75%, respectively [232]. A validation study with alternative sEV-purification techniques after sampling sEVs from peripheral or portal vein blood has reported a drastically reduced sensitivity of 64%, whereas the specificity was still at 90%. Nevertheless, this was more sensitive than fine-needle biopsy and the currently used clinical tumor marker CA19-9 [238]. However, another attempt as part of a study using ELISAs to detect sEVs identified no significant difference in glypican-1 for PDAC patients with respect to benign pancreatic conditions. Thus, further validation and standardization of sEV-purification as well as detection methods is vitally required to allow for routine clinical use of sEVs as diagnostic and prognostic biomarkers [239]. However, sEV-based analysis of biomarkers also has advantages. The nanovesicles protect proteins from proteolytic cleavage and prevent degradation of enclosed nucleic acids [30]. This is why a large number of miRNAs, but also other ncRNAs, in sEVs have been characterized as biomarkers for prognosis and treatment response during tumor progression and metastasis (see Table 2), e.g., mir-21 in sEVs has been associated with lymph node metastasis in PDAC [240], bone metastasis in BC [241], tumor spinal/ventricle metastasis in glioma [242], lymph node metastasis in laryngeal squamous cell carcinoma (LSCC) [243], metastasis in general in ESCC [243], as well as in multi-miR panels with peritoneal metastasis of GC [244], and metastasis in PC [245] or CRC [246]. In addition to RNA, sEV-DNA was also described as a biomarker for the detection of cancer-specific mutations, since sEV-DNA fragments were shown to stochastically represent the entire genome of cancer cells, including mitochondrial DNA [247,248]. Several studies indicated that PDAC-sEVs could be used to probe the mutational landscape of tumors, e.g., for KRAS or TP53 [17,18,249], and an increased mutational allelic frequency in the pool of sEV-DNAs was even correlated with poor prognosis and survival [18,249]. Moreover, to further increase specificity and sensitivity during EV analysis of biomarkers, a strategy for the tumor-specific enrichment of sEVs has been developed [250] for PDAC. Here, a panel of six surface markers on sEVs has been identified to immuno-purify PDAC-specific sEVs after liquid biopsy, thus enabling a more sensitive detection of mutated KRAS alleles [251]. Similar strategies may be applied to enrich sEVs from other cancer entities and different prognostic cargos. As shown in Table 2, nucleic acids, and in particular ncRNAs, in sEVs have a great potential to act as biomarkers for tumor metastatic behavior.

In addition to a role as biomarkers, sEVs have been proposed as therapeutic vehicles, but practical applications are still in their early development phase. sEVs have been explored as novel drug delivery agents due to their inherent non-toxic, biodegradable properties and their ability to cross endogenous barriers, such as the blood–brain barrier [252]. This was demonstrated using engineered sEVs loaded with siRNAs, which targeted the central nervous system (CNS) by expressing Lamp2-RVG on their surface to specifically knockdown genes in the CNS after systemic administration [253]. Another promising study by Kamerkar et al. in 2017 modified sEVs from fibroblast-like mesenchymal cells with siRNAs or shRNAs against mutated and wild-type KRAS^G12D^ (iExosomes), and the treatment of mice with PDAC tumors in a Kras^G12D^ background demonstrated a significant reduction in tumor size, compared to the control. Interestingly, tumors treated with iExosomes also showed superior size reduction with respect to liposomes loaded with the same cargo [254]. A corresponding clinical study is currently on the way as part of a Phase I trial in PDAC patients with a KRAS^G12D^ mutation (NCT03608631). Cancer progression and metastasis in different cancer entities is often associated with downregulation of tumor-suppressor miRNAs. The re-introduction of such miRNAs as EV-based therapies may thus reduce tumor proliferation and invasion. For efficient treatment, the mode of delivery is also a crucial factor, e.g., intra-tumoral injection of miR-335-containing EVs caused cancer inhibition in hepatocellular carcinoma xenograft models [255]. EVs for therapeutic approaches can be obtained from different sources, such as fibroblasts [256], stromal cells [257], red blood cells [258] and umbilical cord stem cells [259], which are subsequently loaded with therapeutic miRNA. NK cell-derived sEVs with miR-186 were used to impair immune escape of neuroblastoma [260], whereas red blood cell-sEVs loaded with miR-125b were shown to inhibit cell proliferation of leukemia and BC in xenografted mice [258]. As discussed earlier, concepts for targeted drug-delivery using engineered sEVs are still in development. It will be necessary to further optimize sEVs to exploit natural tropisms for specific organs and cell populations, or modify sEVs with artificial targeting constructs to increase specificity and uptake in recipient cells. In addition, strategies for loading of sEVs with cargos, e.g., specific siRNAs by electroporation [261], have to be adapted and optimized to increase efficacy for future clinical applications.

## 9. Conclusions and Perspectives

**Figure 2:**
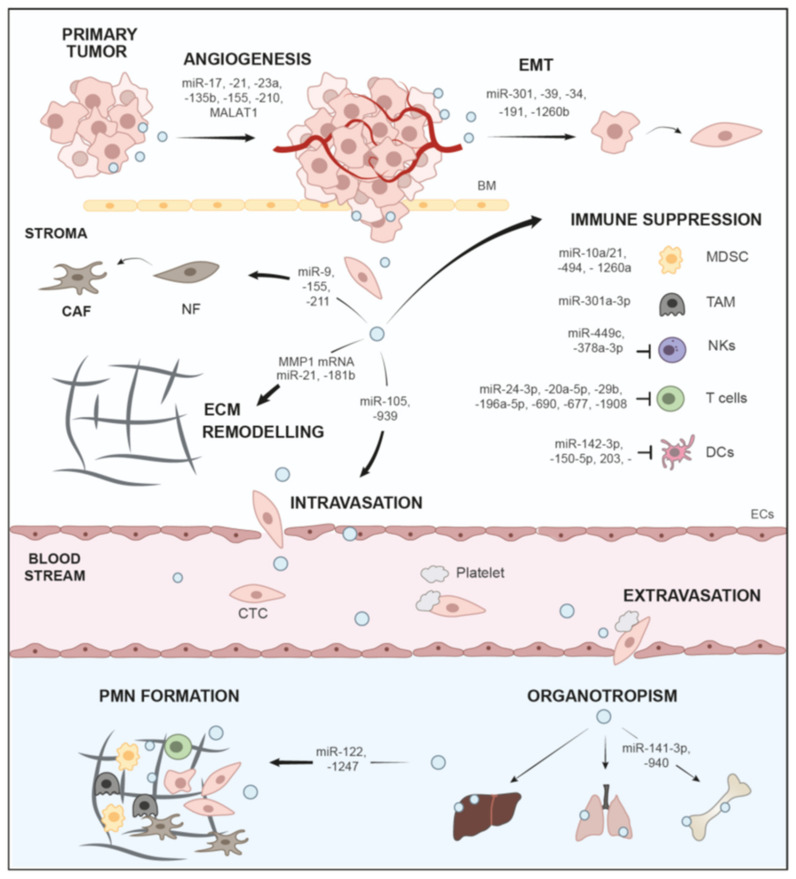
Functions of sEVs and ncRNA cargos in the metastatic cascade.

Here, we have discussed the role of sEVs and the important major cargo class, nucleic acids, in the progression of tumors through different aspects of the metastatic cascade (Figure 2, Table 1), as well as their role as biomarker platforms and vehicles for treatment (Table 2). Tumors release large quantities of sEVs with an altered cargo profile, and this is directed by genetic alteration as well as environmental cues, such as low pH or hypoxia (Section 1 and Section 2). Oncogenes, e.g., mutated KRAS, not only trigger activation of ESCRT and ceramide synthesis pathways to drive enhanced sEV-release from tumor cells, but also change the sEV miRNA content, enabling oncogenic transfer and metabolic reprograming (Section 2.3). Thus, it would be interesting to systematically assess how cancer-relevant genes and mutations affect sEV secretion as well as alterations of cargo content. Nucleic acids, in particular ncRNA cargo, such as miRNAs are vital cargos for the education of recipient cells during metastasis (Table 1). Extensive research in the last years has indicated that tumor sEVs and sEV-based crosstalk with other cell populations in the TME influence local invasion of tumor cells by facilitating EMT, ECM remodeling, stroma reprogramming, immune evasion and angiogenesis (Section 3 and Section 4). In addition, sEVs have been implicated in the establishment of distant organ-specific PMNs with a pro-inflammatory microenvironment (Section 7, Figure 2, Table 1). The intercellular communication network enabled by sEVs, as well as their systemic distribution, are vital factors in determining whether tumor cells successfully metastasize. It has to be noted, however, that the same sEV-cargos, in particular miRNAs, may have different functions dependent on the investigated cancer entities, e.g., miR-21 drives various routes of metastasis, when compared to BC, PDAC or glioma [240,241,242]. Nevertheless, ample evidence has indicated that circulating sEVs can be employed as effective biomarker platforms for diagnosis or prognosis (Section 8, Table 2). sEVs are released in all body fluids and are thought to generally reflect the state of their parental cells [22]. Multiple liquid biopsy studies have indicated that sEVs, and in particular miRNAs, can be used as biomarkers for various cancer subtypes, disease progression, metastasis as well as treatment response (Section 8, Table 2). Moreover sEV-DNA fragments, which statistically cover the entire genome and mutational landscape, were used to assess mutated genes in tumors as prognostic markers (Section 8). sEVs are ideal vehicles in this context, since they protect nucleic acid cargos from degradation. However, there are also caveats, which have so far prevented the broad adoption of sEV profiling in the clinical routine. Different isolation techniques and detection methods have yielded drastically different results, and thus rigorous standardization will be required to promote clinical sEV analysis (Section 8). Another issue might be the heterogeneity of sEVs in liquid biopsy samples, which are not only derived from tumor cells but many other cell types [251]. Thus, to increase specificity and sensitivity, first, steps towards immuno-enrichment of tumor-specific sEVs have been conducted by identifying and utilizing a panel of surface markers to enrich PDAC-derived sEVs [251]. Interestingly, sEVs can also be engineered as vehicles for delivery of therapeutic agents, such as siRNAs and miRNAs to target cancer cells in order to downregulate oncogenes or tumor-promoting factors. For example, transfer of miR-122 was shown to induce chemosensitivity in HCC [280], whereas sEVs loaded with siRNAs against mutated KRAS^G12D^ impaired PDAC tumor growth in mice [254]. The latter study has even resulted in a first phase I clinical trial [254]. However, research on therapeutic sEVs is only at the beginning. Once established and transferred into the clinic, these approaches have immense potential and will open up new avenues to promising treatment options, as shown for sEVs loaded with drugs, such as paclitaxel or doxorubicin [281,282,283]. A major necessity for the design of new sEV-based therapeutic options is improved understanding of molecular mechanisms for sEV-mediated tumorigenesis and metastasis. Additionally, technical issues need to be optimized, such as modification of sEV with cell-specific targeting constructs [284,285], or how natural tropisms of different sEV populations may be exploited to further target and improve uptake in tumor cells [15,286,287]. Nevertheless, sEV research over the last years has greatly contributed to a better understanding of the complex mechanisms that drive tumor progression and metastatic dissemination, and these studies will hopefully help to translate sEV-based applications into clinical use.

## Figures and Tables

**Table 1:** Origin and biological effects of sEV-derived ncRNA.

Step	Molecule	Cell of Origin	Effect	Ref.
EMT	miR-301a	GBM, PC	Activation of Wnt/β-catenin signaling, suppression of TCEAL7, p63 and E-cadherin	[89,124]
	miR-92a	NSCLC	Activation of PTEN/PI3K/AKT	[90]
	miR-191	Melanoma	Activation of MAPK signaling	[14]
	miR-1260b	LC	Activation of Wnt/β-catenin signaling via inhibition of sFRP1 and Smad4	[91]
	miR-181d-5p	CAFs in BC	Suppression of CDC2 and HOXA5	
	miR-23a	NSCLC	Induction of TGFβ signaling and inhibition of E-cadherin	[125]
	miR-21 miR-143 miR-378e	CAFs in BC	Upregulation of NANOG, SOX2, SNAIL and ZEB	[126]
	miR-499a-5p	LC	Activation of mTOR signaling	[127]
	miR-19b-3p	CCRCC	Downregulation of PTEN	[127]
	miR-382-5p	CAFs in OSCC	Upregulation of ß-catenin and N-cadherin	[128]
ECM degradation	miR-21	CRC, EC	MMP activation via inhibition of PDCD4 and TIMP-3	[101,102,104]
	miR-181b	HCC, ESCC	MMP activation via inhibition of TIMP-3	[105,106]
	MMP1 mRNA	OC	Enhanced MMP1 expression in recipient cells	[100]
	miR-382-5p	CAFs in OSCC	Upregulation of MMP-9 and MMP-3	[128]
Anoikis resistance	miR-210	CRC		[92]
	miR-222-3p	NSCLC	Inhibition of SOCS3	[93]
Stroma	miR-9	BC	CAF formation	[121]
	miR-211	Melanoma	CAF formation via MAPK activation	[12]
	miR-155	PDAC	CAF formation via downregulation of TP53INP1	[122]
Immune modulation	miR-24-3p, miR-891a, miR-106a-5p, miR-20a-5p, miR-1908	NPC	T-cell exhaustion via downregulation of the MARK1 signaling pathway	[129]
	miR-690, miR-677, miR-29b	Melanoma	CD4+ T-cell apoptosis, increase of caspase-3, caspase-7 and caspase-9, downregulation of BCL-2	[130]
	circUHRF1	HCC	NK dysfunction via inhibition of IFN-γ and TNF-α secretion	[131]
	miR-301a-3p	PDAC	M2 macrophage polarization via activation of PTEN/PI3Kγ signaling	[132]
	miR-378a-3p	Various cell lines	Decreased NK cytotoxicity via inhibition of granzyme B	[133]
	miR-212-3p	PDAC	DC dysfunction via PFXAP inhibition and compromised MHCII expression	[134]
	miR-150-5p, miR-142-3p	T_regs_	DC exhaustion	[135]
	miR-203	PDAC	DC dysfunction via TLR4 inhibition	[136]
	miR-18a	CeC	T-cell exhaustion, suppression of PTEN, WNK2 and SOX6, and enhanced PD-L1 levels	[137]
	miR-23a	HCC	T-cell exhaustion, suppression of PTEN and enhanced PD-L1 levels	[138,139]
	miR-10a, miR-21	Glioma	MDSC expansion by targeting RAR and PTEN	[140]
	miR-1246	Mutant p53 cancer cell lines	M2 macrophage polarization via targeting TERF2IP	[141,142]
	Let7	Hypoxic tumor cells	M2 polarization and metabolic reprogramming in macrophages via suppression of AKT-mTOR signaling	[143]
	miR-29a-3p and miR-21-5p	TAMs in OC	Suppression of STAT3 in CD4 T-cells and corresponding T_reg_ induction	[144]
	miR-125b-5p	Melanoma	M2 macrophage polarization via downregulation of LIPA	[145]
	miR-27a-3p	BC	PD-L1-mediated immune evasion via targeting MAGI2/PTEN/PI3K signaling	[146]
Intra- vasation	miR-939	BC	Downregulation of VE-Cadherin in ECs	[147]
	miR-105	BC	Downregulation of ZO-1 in ECs	[148]
	miR-181c	BC	Destruction of blood–brain barrier and promotion of brain metastasis via downregulation of PDPK1	[149]
Angio- genesis	miR-17-5p	NPC	Promoting angiogenic activity in ECs via AKT/VEGF-A expression	[115]
	Mir-155	GC	Suppression of FOXO3a and c-MYC to enhance the expression of VEGF in ECs	[150]
	miR-27a	PDAC	Proliferation of ECs via inhibition of BTG2	[13]
	miR-135b, miR-210, miR-21, miR-23a	Hypoxic tumor cells	Induction of blood vessel formation	[151,152,153]
	MALAT1	OC	Pro-angiogenic gene expression in HUVECs	[154]
	miR-205	OC	Promotes angiogenesis in ECs via PTEN-AKT signaling	[127]
	lncRNA-Ccat2	Glioma	Proliferation of ECs via upregulation of VEGF-A and TGFβ. Inhibition of apoptosis by targeting Bax and caspase-3	[155]
	lncRNA-Pouf3	Glioma	Pro-angiogenic gene expression in ECs	[155]
Lymphangiogenesis	miR-221-3p	CeC	Expansion of lymphatic vessels via downregulation of VASH	[156]
	LNMAT2	Bladder cancer	Growth of lymphatic vessels via upregulation of PROX1	[157]
Organo- tropism	miR-141-3p	PC	Bone metastasis via increased osteoblast activity	[15]
	miR-940	PC, BC	Bone metastasis via increased osteoblast activity	[158]
PMN	miR-122	BC	Suppression of glycolytic enzymes in non-tumor cells	[116]
	miR-1247-3p	HCC	CAF formation	[159]
	miR-451	CAFs in ESCC	Pro-tumor PMN formation	[160]
	miR-25-3p	CRC	Enhanced vascular permeability and angiogenesis in PMN via targeting KLF2 and KLF4	[161]

Abbreviations: BC: breast cancer; CCRCC: clear cell renal cell carcinoma; CeC: cervical cancer; CRC: colorectal cancer; ESCC: esophageal squamous cell carcinoma; GBM: glioblastoma; GC: gastric cancer; HCC: hepatocellular carcinoma; LC: lung cancer; NPC: nasopharyngeal carcinoma; NSCLC: non-small-cell lung cancer; OC: ovarian cancer; OSCC: oral squamous cell carcinoma; PC: prostate cancer; PDAC: pancreatic ductal adenocarcinoma; TAM: tumor-associated macrophage; CAF: cancer-associated fibroblast; PMN: pre-metastatic niche.

**Table 2:** sEV-ncRNA cargo used as diagnostic and prognostic biomarkers in cancer.

Cancer	sEV Cargo	Source	Diagnostic/Prognostic Value	Reference
CRC	miR-92a-3p	Serum	Liver and lung metastasis	[262]
	miR-193a	Plasma	Liver metastasis	[47]
	miR-25-3p	Serum	Metastasis (Liver and lung metastasis in mice; involved PMN formation)	[161]
	miR-141-3p miR-375	Plasma	Liver metastasis	[263]
	miR-21-5p	Plasma	Liver metastasis	[264]
	lncRNA CRNDE-h	Serum	Regional lymph node and distant metastasis	[265]
	miR-19b, miR-21, miR-222, miR-92a	Serum	Metastasis	[246]
	Low miR-548c-5p	Serum	Liver metastasis	[266]
PC	miR-141 miR-375	Serum	Metastasis	[267,268]
	Low miR-636 High miR-21 High miR-16 High miR-142-3p High miR-451	Urine	Metastasis	[245]
	miR-1246	Serum	Metastasis	[269]
GC	miR-21 miR-1225-5p	PLF	Peritoneal metastasis	[270]
	miR-21-5p miR-92a-3p miR-223-3p miR-342-3p	PLF	Peritoneal metastasis	[244]
	miR-423-5p	Serum	Lymph node metastasis	[271]
	miR-10b-5p	Plasma	Lymph node metastasis	[272]
	miR-101-3p	Plasma	Ovarian metastasis	[272]
	miR-143-5p	Plasma	Liver metastasis	[272]
OC	miR-200b miR-200c	Serum	Lymph node metastasis	[273]
ESCC	miR-21	Serum	Metastasis	[274]
HCC	miR-665	Serum	Metastasis	[275]
	miR-1247-3p	Serum	Lung metastasis	[159]
LSCC	miR-21	Serum	Lymph node metastasis	[243]
Glioma	miR-21	CSF	Tumor spinal/ventricle metastasis	[242]
BC	miR-105	serum	Distant metastasis	[148]
	miR-21	Serum	Bone metastasis	[241]
NSCLC	circSATB2	Serum	Lymphatic metastasis	[276]
PDAC	miR-17-5p	Serum	Metastasis	[277]
	Circ-IARS	Plasma	Tumor-node metastasis and liver metastasis	[278]
	miR-21	Plasma	Lymph node metastasis	[240]
Melanoma	miR-17 miR-19a miR-21 miR-126 miR-149	Plasma	Metastasis	[279]

Abbreviations: BC: breast cancer; CRC: colorectal cancer; ESCC: esophageal squamous cell carcinoma; GC: gastric cancer; HCC: hepatocellular carcinoma; LSCC: Laryngeal squamous cell carcinoma; NSCLC: non-small cell lung cancer; OC: ovarian cancer; PC: prostate cancer; PDAC: pancreatic ductal adenocarcinoma; CSF: cerebrospinal fluid; PLF: peritoneal lavage fluid.
